# Single molecule and multiple bond characterization of catch bond associated cytoadhesion in malaria

**DOI:** 10.1038/s41598-017-04352-x

**Published:** 2017-06-23

**Authors:** Ying Bena Lim, Juzar Thingna, Jianshu Cao, Chwee Teck Lim

**Affiliations:** 1 Department of Biomedical Engineering, National University of Singapore, Singapore, 117583 Singapore; 2 Singapore-MIT Alliance for Research and Technology (SMART) Centre, Infectious Diseases IRG, Singapore, 138602 Singapore; 3 Department of Chemistry, Massachusetts Institute of Technology, Cambridge, 02139 USA; 4 Mechanobiology Institute, National University of Singapore, Singapore, 117411 Singapore

## Abstract

The adhesion of malaria infected red blood cells (iRBCs) to host endothelial receptors in the microvasculature, or cytoadhesion, is associated with severe disease pathology such as multiple organ failure and cerebral malaria. Malaria iRBCs have been shown to bind to several receptors, of which intercellular adhesion molecule 1 (ICAM-1) upregulation in brain microvasculature is the only one correlated to cerebral malaria. We utilize a biophysical approach to study the interactions between iRBCs and ICAM-1. At the single molecule level, force spectroscopy experiments reveal that ICAM-1 forms catch bond interactions with *Plasmodium falciparum* parasite iRBCs. Flow experiments are subsequently conducted to understand multiple bond behavior. Using a robust model that smoothly transitions between our single and multiple bond results, we conclusively demonstrate that the catch bond behavior persists even under flow conditions. The parameters extracted from these experimental results revealed that the rate of association of iRBC-ICAM-1 bonds are ten times lower than iRBC-CD36 (cluster of differentiation 36), a receptor that shows no upregulation in the brains of cerebral malaria patients. Yet, the dissociation rates are nearly the same for both iRBC-receptor interactions. Thus, our results suggest that ICAM-1 may not be the sole mediator responsible for cytoadhesion in the brain.

## Introduction

Of the five *Plasmodium* (*P.)* genus parasites known to cause malaria in humans, *P. falciparum* contributes most to severe disease and deaths^1, 2^. Infection is initiated when an infected female *Anopheles* mosquito injects sporozoites into the human skin during a blood meal^3, 4^. After traveling to and replicating within liver cells, merozoites are then released into the circulatory system where they invade the red blood cells (RBCs). These merozoites then undergo asexual development from ring to trophozoite to schizont stages within 48 hours. At the end of each cycle, merozoites are released by the schizonts to invade even more uninfected RBCs. Clinical symptoms of malaria – such as chills and fever – present themselves during this asexual blood cycle. To escape the host’s splenic clearance system which removes damaged or aged erythrocytes, the parasite employs a sequestration strategy^5, 6^. This process involves the adhesion of parasite-exported proteins, known as ligands, expressed on the infected RBC (iRBC) membrane to receptors on host cells^3^. iRBCs can either bind to endothelial cells^7^, form rosettes with uninfected RBCs^8, 9^, or cluster amongst themselves via platelet interactions^10^. Sequestered iRBCs occlude microvasculature^11, 12^, induce the release of damaging inflammatory mediators^13^, and cause disruptions to host metabolism^14^. Cerebral malaria, a lethal manifestation of *P. falciparum*, occurs when iRBCs sequester in the microvasculature in the brain^15^.

In a histological study conducted by Turner *et al*., endothelial activation in fatal cases of cerebral malaria was investigated^16^. Results show that two endothelial receptors, Intercellular Adhesion Molecule 1 (ICAM-1) and Cluster of Differentiation 36 (CD36), were present in high amounts in the liver, spleen, kidney, lung and muscle vasculature of infected cases. However, only ICAM-1 was highly expressed in the brains of infected cases^16^. Another clinical study noted a positive correlation between ICAM-1 binding ability and cerebral malaria^17^. Flow assays later demonstrated that iRBCs predominantly roll on ICAM-1 coated substrates but form static adhesions with CD36^18^. These observations led to the hypothesis that ICAM-1 initiates rolling while CD36 stabilizes the iRBC thereafter^18, 19^. Consequently, studies have been conducted to use the soluble forms of these two endothelial receptors to block parasitic domains on the iRBC membrane. CD36 peptides were found to be able to block cytoadhesion of iRBCs as expected^20^. Intriguingly, iRBC adhesion to ICAM-1 is not always affected in the presence of soluble ICAM-1^21^.

These findings led us to raise several questions about ICAM-1 mediated sequestration in cerebral malaria: Why does ICAM-1 mediate the rolling of iRBCs? Why is soluble ICAM-1 unable to prevent iRBC adhesion unlike soluble CD36? What is the strategy employed by the parasite to sequester in ICAM-1 upregulated cerebral microvasculature? Here, we utilize a biophysical approach to tackle these questions. The binding of iRBCs to ICAM-1 are first probed at a single molecule level to provide us with a fundamental understanding of iRBC-ICAM-1 interactions. Next, to translate these single bond observations to a cellular level involving multiple bonds, we conducted flow experiments to study how clusters of bonds behave. Theoretical modeling was then employed to extract association and dissociation parameters of iRBC-ICAM-1 interactions for quantitative analysis. As a comparison, the same studies were repeated for iRBC-CD36 bonds. Results from these experiments can help us better understand the strategies employed by *P*. falciparum parasites to enhance iRBC sequestration in the brain.

## Results

### Single Bond Force Spectroscopy

We first utilized force spectroscopy to study iRBC-ICAM-1 bonds at the single molecule level. Since the most basic form of interaction was studied, results obtained were considerably less complex to analyze^22^. There are three different types of interactions that can be formed between a receptor-ligand pair: slip bonds, catch bonds and ideal bonds. A slip bond is one which breaks at a faster rate when an external force is applied to it^23^. Such bonds are typically formed in most biomolecular interactions such as aptamers^24^ and cell adhesion proteins^25^. A catch bond, on the other hand, is one that takes an increasing amount of time to dissociate when subjected to an increasing force^23^. Leukocytes can form catch bonds via L-selectin with P-selectin glycoprotein ligand-1 (PSGL-1), enabling them to roll along the vasculature above a shear threshold^26^. FimH on *Escherichia coli* bacteria can also bind to mannose via catch bonds to adhere in a “stick-and-roll” manner^27^. Last but not least, the lifetime of an ideal bond is independent on the force acting on it^23^. This bond was recently observed experimentally by Rakshit *et al*. in e-cadherin adhesion^28^. Prior force spectroscopy experiments on CD36-iRBC interactions suggest that it forms a slip bond with iRBCs^29, 30^. Considering that iRBCs were observed to remain adhered to ICAM-1 surfaces at high wall shear stress^18^, we hypothesize that iRBCs bind to ICAM-1 via catch bonds.

**Figure 1:**
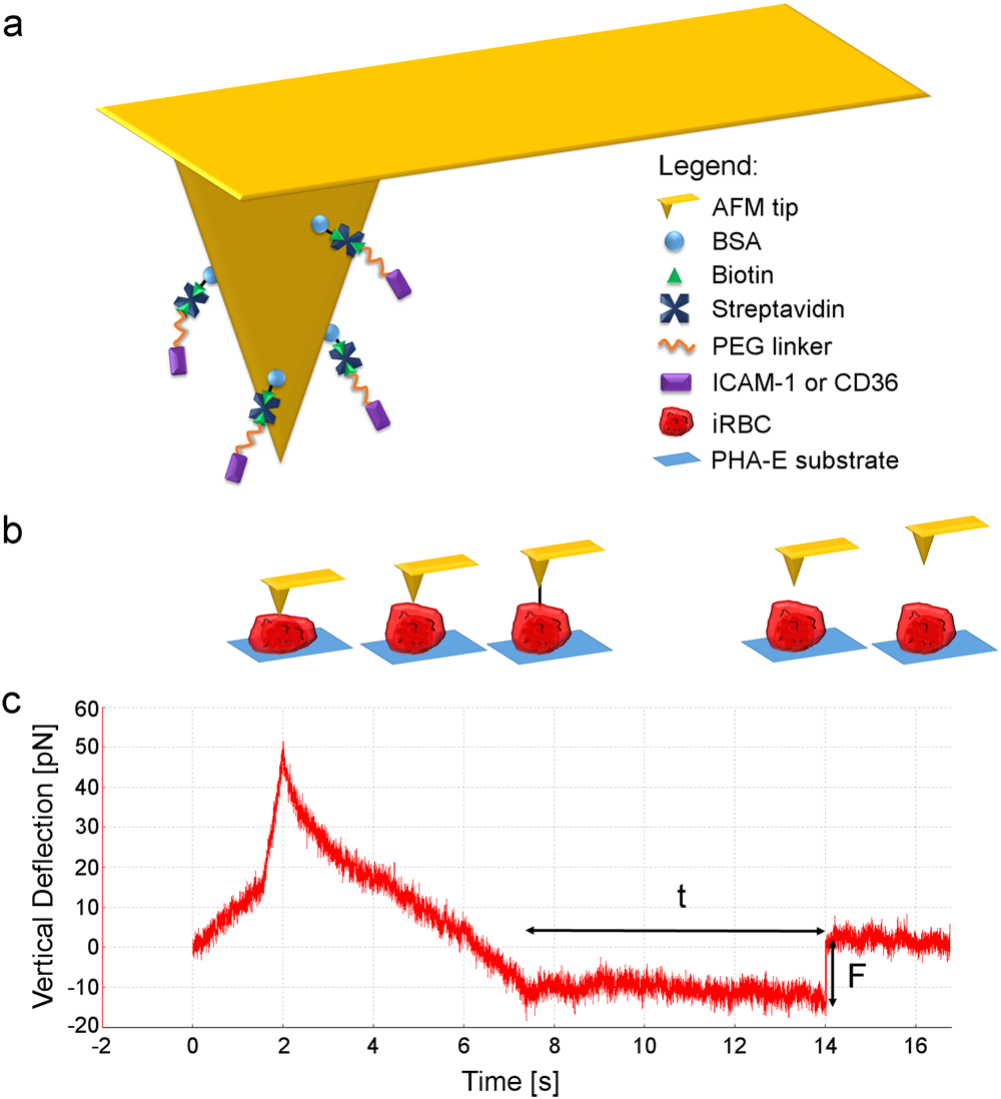
Single bond force spectroscopy experiment. Panel a: Functionalization of AFM tip. Panel b: Schematic of force spectroscopy protocol corresponding to the time scale in panel c. Panel c: Force curve depicting the dissociation of a single bond. Lifetime t and breaking force F of a single bond can be measured as indicated by the arrows.

Bio-Lever atomic force microscope (AFM) tips were functionalized using a protocol similar to that developed by Rakshit *et al*.^28^ (Fig. 1a). Briefly, it involves biotin-streptavidin interactions as well as the use of a polyethylene glycol (PEG) linker. Human recombinant ICAM-1 was then bound to the free end of the PEG linker. The functionalized tip was subsequently used to probe each late stage iRBC in a grid pattern. As depicted in Fig. 1b, each cycle involves contacting the iRBC before retracting to a constant set point. The tip was held there for 10 s for any formed bonds to break. Thereafter, it was further retracted to break any remaining bonds. Only curves with one breaking event during the 10 s constant force period were analyzed by measuring the breaking force and bond lifetime (Fig. 1c). For most adhesion events to be mediated by single bonds, it is necessary to maintain a binding frequency that is less than 20%^31^. In addition to optimizing the concentration of receptors functionalized on the AFM tip, the contact time and approach force were adjusted during the experiment to ensure low adhesion frequency. Numerous measurements over a range of forces were obtained^22, 32^ to account for the random nature of the bond breaking process^33^. The same process was repeated with CD36 for comparison.

Data obtained from both iRBC-ICAM-1 and iRBC-CD36 interactions were pooled and binned into bin widths of 1–2 pN. Considering that our experiments only involve the breaking of single bonds, the probabilistic model for small system kinetics^34^ can be simplified to give

1 $$\frac{dP}{dt}=-{k}_{off}{P}^{m}.$$

**Figure 2:**
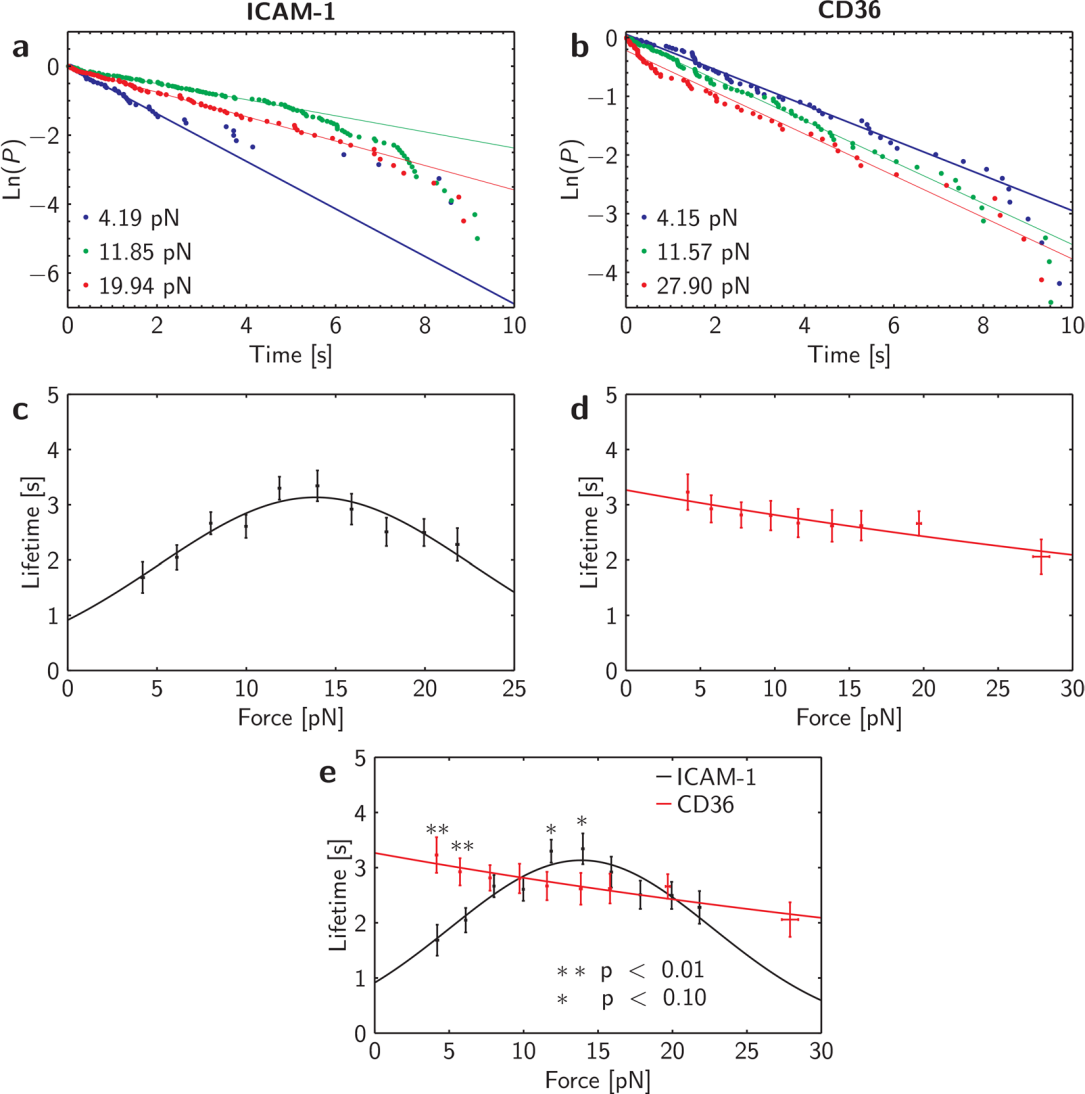
Single bond force spectroscopy results. Panels a and b: Survival probability plots of iRBC-ICAM-1 and iRBC-CD36 bonds respectively. Panels c and d: Lifetime against force graphs of iRBC-ICAM-1 and iRBC-CD36 interactions fitted to the catch and slip bond models. Lifetimes are represented as mean ± SEM. Panel e: Combined plots of panels c and d. Student’s t-test was conducted to compare the lifetimes of the two interactions within each force bin (**p < 0.01, *p < 0.1).

The above equation describes the rate of change of probability *P* of finding a bond between the iRBC and receptor on the AFM tip at time *t*. The dissociation rate is given by the constant *k*_*off*_ while *m* is the order of the kinetics. Assuming that a first order kinetics is involved in the iRBC interaction with ICAM-1 and CD36, this equation can then be analytically solved to obtain

2 $$P=\frac{{e}^{-{k}_{off}t}}{Z},$$

where *Z* is a normalization factor. To ascertain this, we plot the natural log of the number of events with a lifetime greater than *t* against *t* to obtain the survival probabilities of the bonds within each force bin. A straight line with a negative gradient is expected for a receptor-ligand interaction with first order kinetics, whereby bond lifetime can then be taken to be the inverse of the dissociation constant *k*_*off*_. Figure 2a and b show the survival probability plots at three forces for iRBC-ICAM-1 and iRBC-CD36 bonds respectively. Considering that the data points generally fit along a straight line, it is likely that both interactions are of first order.

It was observed that a few points diverged from the trend and they occur mostly at longer lifetimes. Reasons for deviation could possibly be due to non-specific binding, thermal drifting or simultaneous breaking of multiple bonds^35^ even though measures have already been put into place. For instance, adding bovine serum albumin (BSA) into the medium or using linkers to functionalize the tip can reduce non-specific binding^23^. Control experiments conducted to measure RBC-ICAM-1 and RBC-CD36 interactions yielded insignificant number of lifetimes. Adhesion frequency between iRBC and each receptor was also deliberately reduced to less than 20% to ensure that most breaking events were due to single bonds^31^. More importantly, each bin contains at least 50 bonds to represent the stochastic nature of bond dissociation in a more accurate manner^36^. Hence, instead of filtering out these seemingly anomalous data, we employ a robust bi-square fitting using Matlab to assign more weight to the points that are closest to the general trend and lesser weight to those further away from the trend. This method was verified to be consistent with the models used to fit the experimental data, further supporting that first order binding kinetics are involved in both iRBC-ICAM-1 and iRBC-CD36 bonds.

After establishing that both interactions were of first order kinetics, we subsequently proceeded to fit our experimental data to existing models. The model describing the dissociation rate *k*_*off*_ for slip bonds was proposed by Bell^37^ and later theoretically supported by Evans and Ritchie^38^ with

3 $${k}_{off}^{s}={k}^{s}{e}^{\beta F{x}_{0}}.$$

In this model, *k* ^*s*^ is the spontaneous dissociation rate i.e. ${k}_{off}^{s}={k}^{s}$ when the constant force *F* acting on the bond is zero. The inverse temperature $\beta =\frac{1}{{k}_{B}T}$, where *k*_*B*_ represents the Boltzmann constant and *T* is the thermodynamic temperature. Finally, *x*_0_ denotes the stress-free bond length.

On the other hand, the dissociation rate for catch bonds were described by Dembo *et al*.^39^ as

4 $${k}_{off}^{c}={k}^{c}{e}^{\frac{\beta }{2}(\kappa -{\kappa }_{ts}){(y-\lambda )}^{2}}.$$

Here, the ligand-receptor bond is considered to be a Hookean spring with stiffness *κ*. The catch bond kinetics undergoes a transition state with stiffness *κ*_*ts*_ and *λ* is the stress-free length of the bond. It is assumed that the ligand-receptor bond obeys Hooke’s law and thus the external stretching force *F* = −(*κ* − *κ*_*ts*_)(*y* − *λ*_*ts*_). Therefore, after appropriately substituting variables, we obtain the dissociation rate in terms of the force as

5 $${k}_{off}^{c}={k}^{c}{e}^{\beta {(F-{F}_{0})}^{2}\xi }.$$

The force *F*_0_ = −(*κ* − *κ*_*ts*_)(*λ* − *λ*_*ts*_) is the characteristic force at which the bond switches from catch to slip bond behavior and $\xi =\frac{1}{2(\kappa -{\kappa }_{ts})}$ is the inverse effective spring constant. Above, *k* ^*c*^ is the dissociation rate when *F* = *F*_0_. Catch bond behavior can also be described by alternate models^40, 41^, of which the two-pathway model can be supported by Kramers’ rate theory^42^. In our study, we do not have a structural basis to elucidate the conformational changes at sub-molecular levels. It is hence impossible for us to ascertain which one of these several models holds for the iRBC-ICAM-1 bond pair^40^. Thus, our goal here was not to validate the type of catch-bond mechanism but rather, to show that the iRBC-ICAM-1 interaction exhibits catch bond behavior. Consequently, we chose the Dembo model described above as it contains the minimum number of independent parameters to describe a Gaussian, which is observed from the experimental data.

Since the iRBCs interact with both receptors to form bonds that obey first order kinetics, Equations (3) and (5) can then be re-written in terms of the lifetime *τ* of the bond as

6 $$\tau =\frac{1}{{k}^{s}}{e}^{-\beta F{x}_{0}}$$

and

7 $$\tau =\frac{1}{{k}^{c}}{e}^{-\beta {(F-{F}_{0})}^{2}\xi }.$$

Graphs of lifetime against force were plotted and fitted into these models to quantitatively understand the parameters involved in the bond kinetics. Lifetimes are represented as mean ± standard error of mean (SEM). As we have hypothesized, iRBCs form catch bonds with ICAM-1 depicting a distinct peak at the force *F*_0_, and slip bonds with CD36 showing a clear exponential decay of the lifetime as a function of the force (Fig. 2c and d). The fitting parameters are summarized in Table 1. By overlaying the lifetime plots of the iRBC-ICAM-1 and iRBC-CD36 interactions in Fig. 2e, we find the lifetime of iRBC-CD36 significantly higher than that of iRBC-ICAM-1 at low forces (<7 pN) and vice versa in the intermediate range of forces (11–15 pN).

**Table 1:** Fitting parameters for AFM and flow assay.

Parameter	Unit	ICAM-1	CD36
Spontaneous dissociation rate, *k* ^*x*^ (AFM)	s^−1^	0.3192	0.3095
Stress free bond length, *x*_0_ (AFM)	nm	—	0.0613
Inverse effective spring constant, *ξ* (AFM)	nm·pN^−1^	0.0263	—
Characteristic force, *F*_0_ (AFM)	pN	13.87	—
Temperature, *T*	K	298	298
Association rate, ${k}_{on}^{x}$ (Flow)	s^−1^	0.133	1.22
Protein cluster size, *N* (Flow)		7	2
Cell radius, *r* (Flow)	μm	3.5	3.5
Viscosity, *η*	Pa·s	0.001	0.001
Effective number of bonds times the width of the rupture area, *σ* · *a* · *b* · *c* (Flow)	μm	15.97	2.39

### Multiple Bond Flow Assay

**Figure 3:**
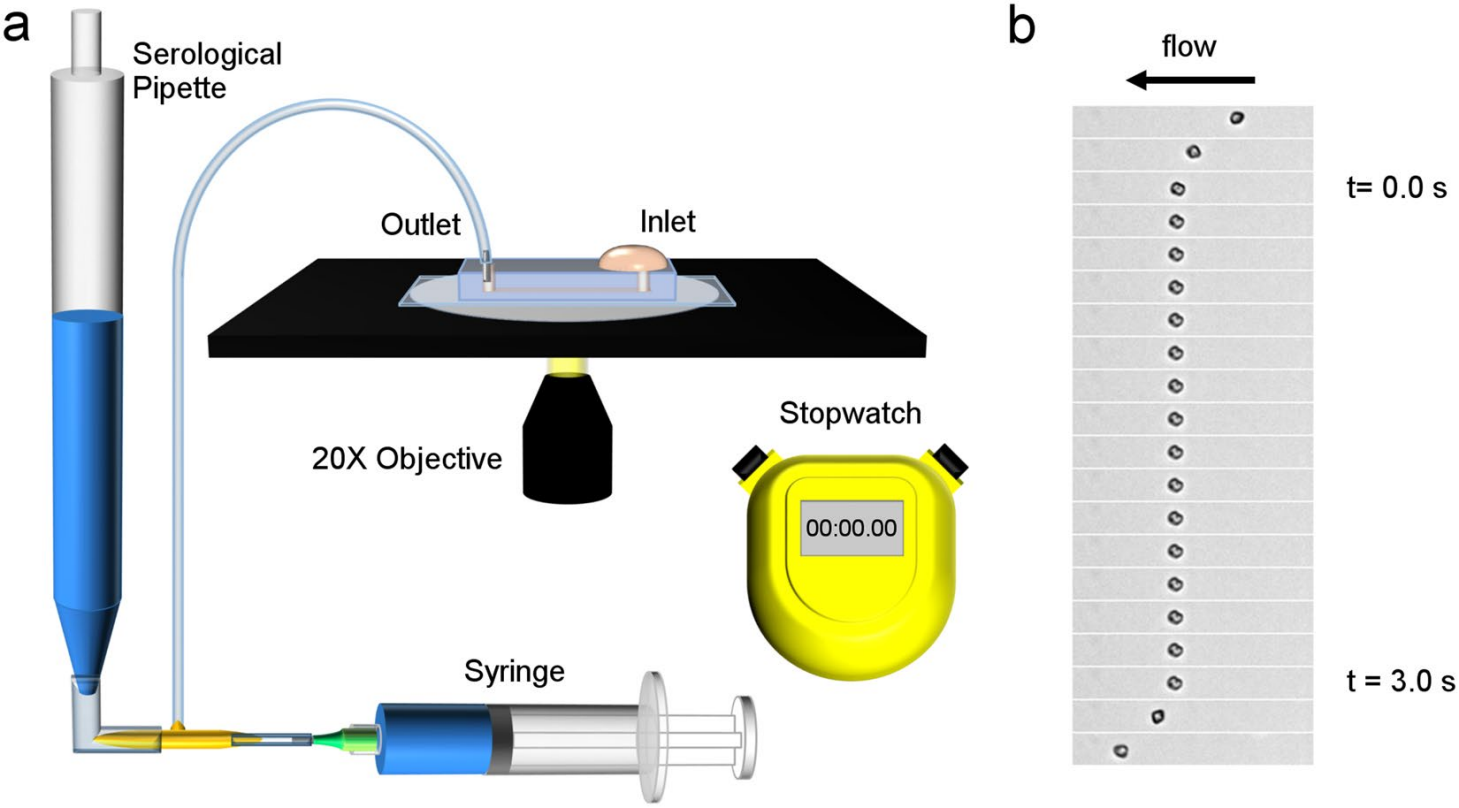
Multiple bond flow experiment. Panel a: Schematic of flow experiment set-up. Panel b: Time lapse image of infected red blood cell adhering in the flow chamber. Each image corresponds to a time difference of 200 ms.

While meaningful insights were gained from the single bond force spectroscopy findings, it is important to note that multiple bonds are formed physiologically. Consequently, we designed a flow assay to study multiple bond interactions between iRBCs and the two endothelial receptors (Fig. 3a). A straight channel with a rectangular cross section was utilized for flow assays (500 µm × 27.6 µm × 1.5 cm). Standard soft lithography techniques were employed in the fabrication of the channels. Prior to the experiment, channels were functionalized either with 50 µg/ml ICAM-1 or 2 µg/ml CD36 using a protocol similar to Xu *et al*.^30^. After ensuring that the serological pipette was vertically aligned, the enriched iRBC suspension was then added at the inlet and the volume of liquid in the serological pipette corresponding to no flow was determined. Thereafter, the liquid volume in the serological pipette was reduced to create a negative pressure at the outlet (a decrease in 0.1 ml corresponds to an increase in shear stress by 0.007 Pa). The center portion along the length of the channel was observed for all experiments. The duration when each cell attached on the substrate was manually measured using a stopwatch. Next, the volume of liquid in the serological pipette corresponding to no flow was determined again before taking more measurements at other shear stresses. The same batch of enriched iRBCs was used to flow in both the ICAM-1 and CD36 coated channels on the same day. Timings were recorded with a precision of 0.1 s to account for human reaction time and the same experimenter did all the recording for consistency.

As illustrated in the time lapse images in Fig. 3b, the measurement begins when the iRBC becomes stationary and stops when the iRBC starts moving again. Since it was unclear if tumbling cells were simply near the substrate or interacting with it, measurements did not involve fast rolling cells interacting with the substrate. Measurements were not done for cases where: 1) an incoming cell came into contact with the stationary cell, 2) the cell adhered for more than 2 min, 3) the cell rolled out of the field of view. Similar to the single bond force spectroscopy experiments, at least 50 data points per shear stress were obtained to account for the stochastic nature of the bond breaking process.

In order to connect the single bond force spectroscopy data to the multiple bond flow experiments, we extended the work of Efremov and Cao^43^ to the catch bond scenario and assumed that within the contact area of the cell, there is a rupture area wherein all the bond kinetics occur. In most of the contact areas, the bonds will be compressed due to the weight of the cell and hence these regions do not significantly contribute to the adhesion kinetics. We also assumed that the cell either attaches or detaches and there is no rolling velocity. Thus, within these assumptions, the lifetime of the cluster can be obtained as (see Methods section for more details),

8 $${\tau }_{N}^{x}=\frac{1}{{k}^{x}}\frac{(N-1)!}{N}{(\frac{{k}_{on}^{x}}{{k}^{x}})}^{N-1}{f}_{N}^{x}.$$

The function ${f}_{N}^{x}$ depends on the type of bond (*x* = *s* corresponds to slip bond and *x* = *c* to catch bond) and the number of bonds *N* in the rupture area. We assume that the association rate ${k}_{on}^{x}$ for both types of bonds is independent of force *F*, consistent with Bell’s assumptions^37^. In the case of slip bonds,

9 $${f}_{N}^{s}=\exp [-Q\beta {x}_{0}\sum _{i=1}^{N}\frac{1}{i}]$$

and in the case of catch bonds,

10 $${f}_{N}^{c}=\exp [-\beta {(\sum _{i=1}^{N}\frac{Q}{i}-{F}_{0})}^{2}\xi ].$$

Above the effective force *Q* acting on each site is given by,

11 $$Q\approx \frac{28.4\pi {r}^{3}}{\sigma abc}{\tau }_{w}.$$

where *σ* is the effective density of ligands or receptors (whichever is dominant), *b* and *c* are the length and width of the rupture area respectively, *r* is the radius of the cell, and $a$ is the width of the contact area (we consider the contact area and the rupture area share the same length *b*). Thus, using Equations (8), (9), (10), and (11) we can obtain the lifetime of the cell. Clearly, the lifetime of the cell depends on the single bond parameters (*k* ^*x*^, *βx*_0_, *βξ* and *F*_0_) thereby connecting the single-bond force spectroscopy to the multi-bond flow assay. The fitting parameters from the flow assay are presented in Table 1.

**Figure 4:**
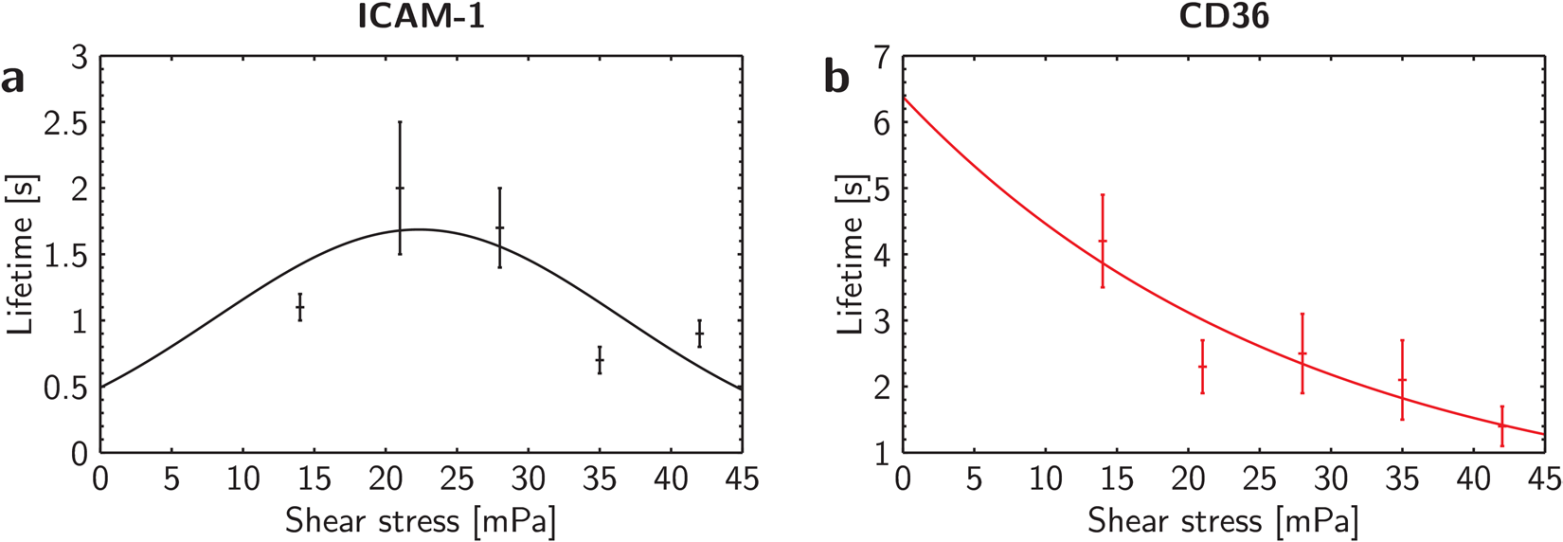
Multiple bond flow experiment results. Panel a: Lifetime against shear stress graphs of iRBC-ICAM-1 interactions fitted to the multiple catch bond model. Panel b: Lifetime against shear stress graphs of iRBC-CD36 interactions fitted to the multiple slip bond model. Lifetimes are represented as mean ± SEM.

As shown in Fig. 4a and b, the plots of lifetime against shear stress for both iRBC-ICAM-1 and iRBC-CD36 interactions obtained from flow experiments follow similar trends as the single bond lifetimes. The solid lines represent the theoretical results obtained by fitting Equations (8), (9), (10), and (11) to the experimental data using a bi-square fit, similar to the single bond force spectroscopy. A characteristic peak is observed at a shear stress of about 21 mPa for ICAM-1 while an exponential decay in lifetime is also present for CD36. Studies have reported enhanced avidity by flow^44, 45^, which could possibly explain the increasing lifetime with increasing shear stress for ICAM-1. However, these findings were unable to explain the part of the curve above 21 mPa where the lifetime decreases. Flow enhanced avidity was also not observed with CD36 even though iRBCs from the same culture were used. In other words, the differences between Fig. 4a and b are most likely accrued to the different interactions between the iRBC and endothelial receptors – catch versus slip bonds – rather than factors arising from the flow.

Even though more bonds are engaged in the iRBC-ICAM-1 interactions than iRBC-CD36 ones, it is evident that the lifetimes of the bonds formed with ICAM-1 are lower than those formed with CD36 over the entire range of flow rates tested. The hypothesis about the cooperation of CD36 and ICAM-1 in stabilizing iRBCs at different shear rates does not seem likely based on these results. This phenomenon can be explained by the much lower *k*_*on*_ for ICAM-1 than CD36. We also noticed the lower binding affinity of the iRBCs to ICAM-1 when optimizing the respective concentrations of receptors used for coating the channel whereby, the concentration of ICAM-1 used to coat the flow channel was 25 times that of CD36. Together, these findings show the importance of considering both association and dissociation parameters in understanding the kinetics of a ligand-receptor pair.

## Discussion

The first question that comes to mind is: what are the implications of iRBC-ICAM-1 catch bond interactions? Firstly, the dynamics of catch bonds can only be observed over a range of forces. It is hence essential for studies involving iRBC-ICAM-1 interactions to be conducted over a range of shear rates for a more accurate representation of adhesive strength and affinity. Next, just like how leukocytes^26^ and bacteria^27^ are able to form catch bonds that mediate rolling and are enhanced in the presence of a force, iRBC-ICAM-1 interactions behave similarly. Our results explain the observations from *in vitro* flow assays that have shown that ICAM-1 mediates rolling of iRBCs as well as the ability of iRBCs to remain adhered to ICAM-1 at high shear rates^18^. Even though the lifetime of individual bonds do not scale linearly with an increase in number of bonds, our force spectroscopy results support the commonly accepted hypothesis that ICAM-1 mediates rolling and adhesion stability at high shear rates while CD36 stabilizes the iRBC at lower shear rates^18, 19^. We further propose that this catch bond interaction sheds light on why soluble ICAM-1 is unable to block adhesion to iRBCs unlike soluble CD36. Bearing in mind that catch bonds have increased lifetimes only when external force is applied, iRBC-ICAM-1 bonds tend to dissociate rapidly when free-floating soluble ICAM-1 interacts with the iRBC due to the absence of forces acting on the receptor-ligand pair. This scenario is unlike the case when ICAM-1 is anchored in position by the endothelial cell, whereby blood flow will exert a force on the iRBC-ICAM-1 bonds. Moreover, high levels of soluble ICAM-1 in the plasma and brain are correlated with the severity of malaria^46, 47^. It is hence advantageous for iRBCs to form catch bonds with ICAM-1 as a sequestration strategy to avoid being affected by the high amounts of soluble ICAM-1 present in the brain.

Interestingly, the subsequent flow experiment and modeling work seemed to suggest that ICAM-1 alone may not be able to account for the accumulation of iRBCs in the brain due to the short adhesion lifetimes as well as low association rates. This discrepancy between the flow results and force spectroscopy findings led us to question the significance of ICAM-1 in contributing to sequestration in cerebral malaria. ICAM-1 upregulation is the only endothelial receptor known to have a positive correlation with cerebral malaria. Yet, the low association affinity between iRBC and ICAM-1 suggests that ICAM-1 may not be able to stabilize the binding for sequestration to occur. Could other receptors like CD36 be involved in cerebral malaria too? Despite having very low levels of CD36 expression in the brain, significant co-localization of sequestration was observed with CD36, ICAM-1 and E-selectin in cerebral vessels^16^. The comparatively higher association affinity of CD36 and our lifetime measurements indicate that it is not necessary for high amounts of this receptor to be present to stabilize the binding of iRBCs. Furthermore, platelets have been shown to bind to endothelial cells and mediate binding of iRBCs, of which CD36 is one of the potential receptors involved^48, 49^. Hence, it is likely that ICAM-1 is not solely responsible for all the sequestration in cerebral vasculature.

The 3D7 strain used in our study was selected for ICAM-1 and may not be an accurate representation of the interactions occurring in cerebral malaria. *P. falciparum* Erythrocyte Membrane Protein-1 (PfEMP1) family comprises antigenically variable proteins expressed on the iRBC membrane that can mediate cytoadhesion to host cells via receptors such as ICAM-1 and CD36^50^. The antigenic diversity of PfEMP1 (regulated by approximately 60 *var* genes per parasite genome^51^) enables the malaria parasite to avoid the host’s immune system and prolong the duration of infection^52^. Based on genetic organization, chromosomal location and strain structure, researchers were able to classify these *var* genes into three groups^53^. However, conflicting results have been reported on the relationship between each group and cerebral malaria^51^. The selection for ICAM-1 may result in a mixture of different groups of PfEMP1 expressed across the cells used in our experiments. As a result, the findings in our study are relevant to iRBC-ICAM-1 interactions in general but may not be specific to those involved in cerebral malaria. Flow experiments were limited to a small bond cluster size, via reducing the receptor concentration for coating the substrate, as well as low shear rates to enable the observation of cells binding and detaching. Furthermore, as we were unable to discern between cells tumbling near the substrate and cells rolling due to interaction with the substrate, our flow experiments only captured a subset of the iRBC-receptor interactions. It is hence noteworthy that our multiple bond data does not involve rolling, which may be essential under physiological conditions.

Nonetheless, our work provides a fresh insight into this longstanding biological problem. The systematic study of iRBC-ICAM-1 bonds from single molecule to multiple bonds enabled us to understand detailed bond kinetics by first probing the fundamental interactions. Such an approach will also be useful in directly determining the nature of other iRBC-receptor interactions that behave like catch bonds under flow conditions. For instance, recent hydrodynamic experiments by Rieger *et al*. have suggested catch bond interactions between VAR2CSA encoded by the iRBC and chondroitin-4-sulfate (CSA) expressed on placental proteoglycans^54^. Computational modeling further empowered us to extract quantitative parameters that can be utilized for comparison with other iRBC-receptor interactions such as iRBC-CD36 bonds as presented in this paper. Moreover, iRBC-receptor dynamics can also be simulated using these experimentally obtained parameters to probe deeper into the interactions in future studies.

## Conclusions

Here, we used a biophysical approach to study iRBC-ICAM-1 interactions at both the single molecule and multiple bond level. Force spectroscopy results showed that ICAM-1 forms catch bonds with iRBCs unlike CD36, which forms slip bonds. Our findings highlighted the importance of conducting flow assays over a wide range of flow rates to fully capture the adhesion of iRBCs to ICAM-1. We were also able to understand why ICAM-1 is able to mediate the rolling of iRBCs as well as propose a reason as to why soluble ICAM-1 is not as effective in preventing iRBC-ICAM-1 adhesion. However, to better understand these interactions in a more physiologically relevant manner, flow experiments were conducted to study multiple bond behavior. We subsequently proposed a computational model that could capture the lifetime of the cells exhibiting catch bond behavior under flow conditions and enable us to extract bond association parameters. Interestingly, while dissociation constants are similar, the association constant for iRBC-ICAM-1 bonds is ten times lower than iRBC-CD36 bonds. This result suggested that ICAM-1 may not be the sole mediator of sequestration in cerebral malaria and it is likely that, despite its low level of expression in brain microvasculature, CD36 is involved too.

## Methods

### Parasite culture

Laboratory adapted *P. falciparum* 3D7 knob- positive strain was used in these experiments^29^. Parasites were cultured *in vitro* with human erythrocytes (informed consent from donors obtained) using a protocol developed by Trager and Jensen^55^. Briefly, malaria culture medium (MCM) was prepared by supplementing RPMI 1640 (Invitrogen) with 0.5% Albumax II (Invitrogen), 50 µg/ml hypoxanthine (Sigma Aldrich), 2 mM L-glutamine (Sigma Aldrich) and 25 µg/ml gentamycin (Gibco Life Technologies). Parasites were cultured at 2.5% hematocrit and incubated at 5% CO_2_, 3% O_2_ and 92% N_2_ at 37 °C. Synchronization of culture was achieved using 5% sorbitol when majority of the infected cells were in the ring stage. Parasitemia was determined by observing Giemsa-stained blood smears under a microscope at 100x magnification.

### iRBC Enrichment

iRBCs in the schizont stage contain paramagnetic iron in the hemozoin, allowing them to be separated from the initial culture using magnetic-activated cell sorting (MACS)^56^. In our experiments, the LD column (MiltenyiBiotec) and SuperMACS II Separator (MiltenyiBiotec) were used to enrich late stage iRBCs. The LD column was first inserted into the separator before rinsing with MCM. Subsequently, the culture suspension was added and subjected to the magnetic field from the SuperMACS II Separator. MCM was added to the column at least thrice to ensure that the uninfected and ring stage erythrocytes were rinsed out of the system. To collect the late stage iRBCs, the column was first removed from the separator and placed in a clean collection tube. 4 ml MCM was added twice to elute all iRBCs in the column. The resultant cell suspension was then centrifuged at 600 g for 5 min before removing the supernatant. Finally, the cell pellet was re-suspended in MCM to an appropriate concentration for experiments. The amount of late stage iRBCs obtained after enrichment is generally above 80%.

### ICAM-1 selection

*P. falciparum* 3D7 parasites were selected for adhesion to ICAM-1. A 22 mm diameter cover slip was first rinsed with 70% ethanol before dipping into de-ionized (DI) water. After the cover slip has been dried, it was placed in a 6-well plate and plasma treated for 10 min. The 6-well plate was subsequently placed in the biosafety cabinet and treated with ultraviolet (UV) radiation for 30 min. 100 μl of 100 μg/ml ICAM-1 protein was added to the cover slip before sealing the plate with parafilm and incubating overnight at 4 °C. 1x Phosphate Buffered Saline (PBS) was used to wash the ICAM-1 cover slip the following day. Next, the surface was incubated with 1% Bovine Serum Albumin (BSA) (Miltenyi Biotec) for 30 min to reduce non-specific binding. The cover slip was rinsed with PBS before adding enriched iRBCs and incubating in the malaria culture incubator for 1 hour. Unbound iRBCs were washed with RPMI for more than five times. Finally, 5 ml malaria culture medium and 250 μl of fresh RBC pellet were added into the well for continuous culture over the next few days. When the selected parasite parasitemia reached more than 5%, a second selection was performed. The expanded culture was aliquoted and frozen for future experiments.

### Force spectroscopy substrate preparation

The substrate for force spectroscopy was prepared by first washing a 30 mm diameter cover slip with ethanol. Thereafter, the cover slip was dipped into DI water and dried in the oven. It was then plasma treated for 10 min before adding 300 μl of 50 µg/ml Phaseolus vulgaris-Erythroagglutinin (PHA-E) (Vector Laboratories). This lectin is known to have high affinity to glycophorin on human erythrocytes^57^. After incubating for an hour, the cover slip was washed thrice with 1 x PBS. Magnetically enriched late stage iRBCs were subsequently added to it and incubated for 30 min at room temperature. Unbound iRBCs were removed by washing the cover slip with MCM thrice. Finally, the cover slip was stuck onto the lid of a 60 mm diameter petri dish using adhesive before filling the lid with 1% BSA in MCM to reduce non-specific adhesion of cellular debris to the atomic force microscope (AFM) tip.

### AFM tip functionalization

Gold coated Bio-Lever AFM tips (Olympus) utilized in force spectroscopy experiments were functionalized using a protocol similar to that developed by Rakshit *et al*.^28^. The tip was first plasma treated for 15 min before incubating with 10 μl of 1 mg/ml BSA-Biotin (Sigma Aldrich) overnight at 4 °C in a humidified chamber. On the following day, the tip was then incubated with 10 μl of 10 µM streptavidin (ThermoFisher Scientific) for 30 min before immersing the tip in 200 µl of 10 mg/ml NHS-PEG4-Biotin (ThermoFisher Scientific) for another 30 min. The tip was subsequently incubated with 10 μl of human ICAM-1 protein (100 µg/ml) and CD36 (66 µg/ml) (Sino Biological Inc.) for an hour. Lastly, additional activated amine groups were blocked by incubating with 1 mg/ml glycine (Sigma Aldrich) for 30 min. Tips were rinsed with 1 × PBS after each incubation step.

### Force spectroscopy

The functionalized Bio-Lever AFM tip was first mounted onto the JPK NanoWizard II and subsequently placed on the stage of the Olympus IX81 microscope. The longer cantilever (100 µm × 30 µm) was then calibrated *in situ* using thermal fluctuation analysis in-built in the JPK SPMControl Software v.4. Spring constants ranged from 2–12 pN/nm. Next, the cantilever was placed above an iRBC which was then probed repeatedly in a grid pattern. In each cycle, the tip of the cantilever approached the cell at a constant speed of 1.5 µm/s and was immediately held there for 50–200 ms to allow for bond formation. Thereafter, it retracted at 250 nm/s to a force between 0–25 pN and maintained at the same force for 10 s. The tip was then retracted at a rate of 2 µm/s to break all remaining bonds before approaching the next location on the iRBC. For most adhesion events to be mediated by single bonds, it is necessary to maintain a binding frequency that is less than 20%^31^. In addition to optimizing the concentration of receptors functionalized on the AFM tip, the contact time and approach force was also adjusted during the experiment to ensure low adhesion frequency.

JPKSPM Data Processing software was utilized to process the raw data. A 1.00 Gaussian smoothing filter was first applied to all the curves to better identify of low force breaking events. Next, the “automatically subtract baseline” function was used to correct the baseline to 0 pN. Only curves with one breaking event during the 10 s constant force period were analyzed by measuring the breaking force and time where force is constant. All the data were pooled and binned into bin widths of 1–2 pN. Each bin contains at least 50 lifetimes.

### Flow channel fabrication

A straight channel with a rectangular cross section was utilized for flow assays. The master mold was made by patterning the 2D image of the channel on a silicon wafer using 27.6 µm thick SU-8 photoresist and standard photolithography methods. This mold was then silanized by vaporizing 60 µl of trichloro (1H,1H,2H,2H-perfluoro-octyl) silane (Sigma-Aldrich) for 8 min before allowing it to settle down on the silicon wafer for 30 min. Subsequently, the polydimethylsiloxane (PDMS) pre-polymer base and cross linking agent (Dow Corning) were mixed 10:1 by weight and poured over the mold. The mold was then left in the desiccator to remove all the bubbles in the mixture before curing in the oven for 2 h at 80 °C. After the PDMS is fully cured, it is released from the mold and cut out the individual channels before punching holes at the inlet and outlet. The underside of the channel and a clean cover glass were then plasma treated before binding them together. Finally, the bonded channels were placed in the oven for 2 h at 80 °C.

### Flow channel functionalization

Flow channels were functionalized using a protocol similar to Xu *et al*.^30^.To prevent the entry of bubbles, the flow channel was first primed with 70% ethanol. The channel was then washed with 1 × PBS before filling it with either 2 µg/ml CD36 or 50 µg/ml ICAM-1. Next, it was sealed in a humidified chamber and incubated overnight at 4 °C. After washing with PBS the next day, the channel was filled with 5% BSA in PBS and incubated for 1 h at room temperature. The channel was then washed with PBS just before the experiment.

### Flow experiment

Prior to the experiment, a spirit level was used to ensure that the serological pipette was vertically aligned. The enriched iRBC suspension was then added at the inlet and the volume of liquid in the serological pipette corresponding to no flow was determined by using the syringe. Thereafter, the liquid volume was reduced by 0.2 ml (equivalent to an increase in shear stress by 0.014 Pa). The center portion along the length of the channel was observed. The duration when each cell attached on the substrate was manually timed using a stopwatch up to 2 min. Next, the volume of liquid in the serological pipette corresponding to no flow was determined again before reducing the volume by 0.3, 0.4, 0.5 and 0.6 ml respectively for further measurements to be taken. The same batch of enriched iRBCs was used to flow in both the ICAM-1 and CD36 coated channels on the same day.

### Modeling the adhesion lifetime in flow

Following the work of Efremov and Cao^43^, we first expressed the shear rate in terms of the force experienced by each bond in the rupture area. We simplified the contact area of the cell as a rectangle with width *b* and length *a*. Within this small contact area, we further assume that there is a rupture area that contains most of the bonds and effectively governs the lifetime of the cell. Thus, the total tension *Q*_*tot*_ experienced by all adhesion sites is:

12 $${Q}_{tot}=K\cdot Q,$$

where *Q* is the effective force acting on each site and *K* = *σbc* is the number of bonds in the rupture area with *σ* being the effective density of receptors or ligands (whichever is dominant). By solving the mechanical equilibrium equations similar to those by Hammer *et al*.^58^ under the assumption that the cell is either attached or detached, i.e., rolling velocity *v* ≈ 0 we obtain,

13 $${Q}_{tot}\approx \frac{28.4\pi {r}^{3}}{a}{\tau }_{w},$$

where *r* is the radius of the cell and *τ*_*w*_ is the shear stress. Therefore, the force *Q* experienced by each bond can be expressed in terms of the shear stress *τ*_*w*_ as,

14 $${Q}_{tot}\approx \frac{28.4\pi {r}^{3}}{\sigma abc}{\tau }_{w}.$$

It is worth noting here that our aim is simpler than that of Efremov and Cao^43^. In their work, they were interested in finding a relation between the cells velocity and the shear rate. In our case we would like to construct a simplistic model relating the shear rate to the tension experienced by a single adhesion bond, when the rolling velocity of the cells *v* ≈ 0. The above description of the tension *Q* is not constrained by the bond behavior and hence could describe catch and slip bonds. Similar to the work of Erdmann and Schwarz^36, 59^ the adhesion of a cluster can be represented as a chain reaction,

$$N\rightleftarrows N-1\rightleftarrows \cdots 2\rightleftarrows 1\to 0,$$

where the right pointing arrow denote the dissociation rate *k*_*i,i−*1_ of going from *i* to *i* − 1 and the left-pointing arrow denotes the association rate *k*_*i−*1, *i*_ of going from *i* − 1 to *i*. The state 0 denotes all bonds being detached in the cluster and hence there is no association rate from state 0 to 1. The average lifetime of a single adhesion site can be obtained by calculating the mean turn-over time^60, 61^ for the chain as,

15 $$\tau (N)=\sum _{i=1}^{N}{\rho }_{i}+\sum _{j=1}^{N-1}[\frac{1}{{\rho }_{j}\,{k}_{j,j+1}}\,\sum _{m=j+1}^{N}{\rho }_{m}],$$

where ${\rho }_{1}=\frac{1}{{k}_{1,0}}$ and ${\rho }_{i}={\prod }_{j=1}^{i-1}{k}_{j,j+1}/{\prod }_{j=1}^{i}{k}_{j,j-1}$. The exact expression for the lifetime Eq. (15) can be further simplified as Eq. (8) when the shear stress *τ*_*w*_ is low (⇒*Q* is low), i.e., ${k}_{i,i+1}\gg {k}_{i+1,i}$, where $i\in [1,N-1]$. This result agrees very well with the results obtained by Erdmann and Schwarz^59^.

### Ethics statement

Blood donation protocol is approved by the National University of Singapore Institutional Review Board. Blood drawing was carried out in accordance with the relevant guidelines and regulations at the National University Hospital Blood Donation Centre. Informed consent was obtained from all donors.
